# Simple Sequence Repeat (SSR)-Based Genetic Diversity in Interspecific Plumcot-Type (*Prunus salicina* × *Prunus armeniaca*) Hybrids

**DOI:** 10.3390/plants11091241

**Published:** 2022-05-04

**Authors:** Brenda I. Guerrero, María Engracia Guerra, Javier Rodrigo

**Affiliations:** 1 Departamento de Ciencia Vegetal, Centro de Investigación y Tecnología Agroalimentaria de Aragón (CITA), Avda Montañana 930, 50059 Zaragoza, Spain; jrodrigo@aragon.es; 2 Instituto Agroalimentario de Aragón-IA2 (CITA-Universidad de Zaragoza), 50013 Zaragoza, Spain; 3 Área de Fruticultura Mediterránea, CICYTEX-Centro de Investigación ‘Finca La Orden-Valdesequera’, A-V, KM 372, Guadajira, 06187 Badajoz, Spain; mariaengracia.guerra@juntaex.es

**Keywords:** pluot, aprium, Japanese plum, apricot, microsatellites, population structure, DAPC

## Abstract

The main objective of many fruit-breeding programs around the world is the release of new cultivars from interspecific hybridizations between species of the *Prunus* genus. Plum × apricot (*Prunus salicina* Lindl. × *Prunus armeniaca* L.) are the most widespread interspecific hybrids, which include plumcots, pluots, and apriums. In this work, 115 accessions of interspecific hybrids from different origins and 27 reference genotypes of apricot and other diploid plum species were analyzed using eight simple sequence repeat (SSR) markers to assess the population structure and current genetic diversity. A total of 149 alleles were obtained, with an average of 19 alleles per locus. The overall polymorphic information content (*PIC*) mean value of SSR markers was 0.81, indicating a high degree of polymorphism of the SSR. The genetic analysis revealed 141 unique genotypes and two synonyms. The unweighted pair group method with arithmetic averages (UPGMA) dendrogram and the population structure with five groups inferred through the discriminant analysis of principal components (DAPC) revealed a clear genetic differentiation between apricot genotypes and the rest of the accessions since the interspecific hybrids clustered with the Japanese plum genotypes. Repeated backcrosses between interspecific hybrids with plum genotypes could be the cause of the higher genetic proximity of the hybrids with respect to plum than with apricot genotypes. This corresponds to the fruit morphology and agronomic behavior observed in most interspecific hybrids, which also resemble plums more than apricots.

## 1. Introduction

The genus *Prunus* comprises 400–430 species and is one of the most diverse genera within the Rosaceae family, with species of great commercial interest such as *Prunus armeniaca* L. (apricot), *Prunus avium* L. (sweet cherry), *Prunus domestica* L. (European plum), *Prunus dulcis* Mill. (almond), *Prunus persica* (L.) Batsch (peach), and *Prunus salicina* Lindl. (Japanese plum) [1]. The close affinity of some species of this genus has led to spontaneous hybrids of *Prunus* that have been cultivated for generations in several regions of the world, such as the hybrids *P. armeniaca* × *Prunus cerasifera* Ehrh., traditionally grown in southwest Asia [2]. These hybrids, also known as ‘black apricots’, are so abundant that they have been classified as a different species, *Prunus dasycarpa* Ehrh. [3]. This genetic proximity between *Prunus* species allows the development of interspecific hybridizations for breeding purposes [4].

Luther Burbank obtained more than 100 cultivars from interspecific crosses between *P. salicina* and at least 15 different diploid plum species between the late 19th and early 20th centuries [5]. The Japanese plum cultivars currently grown do not belong to a pure species but are hybrids derived from the first hybridizations carried out by Burbank and the subsequent ones carried out by modern breeding programs [2]. Burbank also obtained hybrids between plum and apricot, which he called ‘plumcots’, combining the terms ‘plum’ and ‘apricot’ [6], introducing the first commercial cultivars at the beginning of the 20th century, ‘Apex’ [7] and ‘Rutland’ [4].

Currently, many fruit-breeding programs around the world use interspecific hybridizations between *Prunus* species for objectives such as self-compatibility and resistance to Sharka disease (*Plum pox virus*, PPV) [8] as well as new attractive fruits with high sugar contents [9]. In these interspecific crosses, *P. salicina* and *P. cerasifera* are usually used as female parents to ensure fruit set and obtain a greater number of offspring [10,11], and *P. armeniaca*, *P. persica*, and *P. avium* as male parents [12]. Plumcots are the most widespread interspecific hybrids, with a number of commercial cultivars available such as ‘Red Velvet’, ‘Royal Velvet’, ‘Flavor Supreme’, and ‘Flavor Queen’ [7]. These hybrids are highly appreciated because they combine the firmness of flesh and the wide range of flowering and ripening periods of the Japanese plum with the aroma and flavor of the apricot [13]. However, the first plumcots were not very productive and were not grown on a large scale [7,12]. In the last decades, a number of cultivars of great commercial interest have been released through back- and inter-crosses between plumcot, apricot, and Japanese plum [13]. In 1991, Zaiger Genetics (Modesto, CA, USA) registered the terms ‘pluot’ and ‘aprium’, derived from combinations of the terms ‘plumcot’, ‘plum’, and ‘apricot’ [9] and, in 2012, the terms ‘pluerry’ (plum × sweet cherry hybrids) by combining the terms ‘plum’ and ‘cherry’ and ‘peacotum’ a complex peach × apricot × plum hybrid [12]. In theory, pluots are the result of crossing plumcot × plum, so the new hybrid is 75% plum and 25% apricot. On the other hand, apriums are obtained from crossing plumcot × apricot, obtaining hybrids that are 75% apricot and 25% plum [7]. However, these are complex hybrids because the plum cultivars used as parents do not belong to a pure species but rather are hybrids of several species of diploid plums [14,15].

Plumcot fruits have a higher content of sugars, such as glucose and fructose, than plums or peaches, although they do not exceed the sugar content of apricots [16]. Pluot fruits have a higher concentration of soluble solids and lower acidity than plums [17], which makes them highly appreciated by consumers. In general, plumcot and pluot fruits have the appearance of Japanese plums, making it difficult for consumers to distinguish between them [9,13].

Although most of the currently grown Japanese plum-type cultivars are actually hybrids [18], modern stone-fruit-breeding programs use the term ‘interspecific hybrids’ to refer to releases derived from crosses between plum and apricot, as well as backcrosses of plumcots with other species of the genus *Prunus* [7]. The release of a large number of new interspecific hybrids has caused the genetic diversity of the currently available plant material to be unknown, since previous works have focused mainly on the identification of Japanese plum cultivars and only a low number of interspecific plum × apricot hybrids has been analyzed [15,19,20,21,22,23]. The assessment of genetic diversity and population structure of these hybrids could be very useful to determine the genealogical origin of complex interspecific hybrids and to evaluate the effects of introgression. For this purpose, SSR markers are one of the most powerful molecular tools for determining the genetic relationships, genetic diversity, and population structure because they are highly informative, polymorphic, codominant, and exhibit transferability among closely related species of the genus *Prunus* [20,21,24,25].

The objective of this work is to assess the genetic diversity and population structure of interspecific hybrids of plum with other *Prunus* species, mainly apricot. For this purpose, 115 accessions of interspecific hybrids, including commercial cultivars and advanced selections, and 27 reference genotypes have been analyzed using eight simple sequence repeat (SSR) markers.

## 2. Results and Discussion

### 2.1. SSR Polymorphism and Genetic Diversity

All the SSR markers used in this study showed correct amplification and turned out to be polymorphic in the analysis of 115 accessions of interspecific plum × apricot hybrids and 27 reference genotypes. The genetic profiles of each accession obtained with the eight SSR markers are included in Table S1 of the Supplementary Materials. The parameters of SSR genetic diversity are summarized in Table 1. A total of 149 alleles were detected in the whole population, ranging from 12 (CPPCT033) to 21 (BBPCT007 and UDP96005) alleles per locus (*N_A_*), with an average value of 19. These values were similar to those reported in previous works in apricot (48 accessions, 20 SSR, *N_A_* = 4.1 [24]; 890 accessions, 25 SSR, *N_A_* = 24.36 [25]) and Japanese plum (47 accessions, 8 SSR, *N_A_* = 13 [22]). However, the values reported herein were higher than those obtained in a previous study of commercial cultivars of apricot (202 accessions, 10 SSR, *N_A_* = 6.3 in recent releases and *N_A_* = 9.3 in commercial releases), which can be related to the genetic bottleneck as a consequence of the use of common parents in breeding programs of apricot [26]. The smaller allele was obtained with the SSR marker UDP6005 (95 bp) and the larger one with CPSCT005 (229 bp). These results confirmed the high transferability among *Prunus* species of the eight SSR markers used in this study and previously observed in other works [15,20,24,25] and the utility of these SSR markers in the interspecific plum × apricot hybrids and progenies.

The polymorphic information content (PIC) ranged from 0.61 (CPPCT033) to 0.90 (CPSCT005), with a mean value of 0.81; therefore, the alleles were considered to be highly polymorphic, showing their usefulness for the study of genetic diversity [27]. The observed heterozygosity values (Ho) ranged from 0.48 (BPPCT039) to 0.85 (pchgms2), with an average value of 0.70, whereas the expected heterozygosity (He) ranged from 0.58 (CPPCT033) to 0.87 (CPSCT005), with a mean value of 0.76. These values were similar to those observed in several plum species such as *P. salicina* (*Ho* = 0.71; *He* = 0.67), *P. domestica* (*Ho* = 0.73; *He* = 0.69), and *P. insititia* (*Ho* = 0.74; *He* = 0.70) [28], indicating that the number of accessions analyzed in this work is a representative sample of the current genetic variability. The heterozygosity (*Ho* and *He*) of the analyzed accessions was lower than that observed in apricot cultivars, a crop in which a decrease in genetic diversity has been observed [26]. The *F*_IS_ values ranged from −0.13 (CPPCT033) to 0.37 (CPSCT026), with a mean of 0.08, indicating that there is no inbreeding in the whole population since values were observed close to zero. The *F*_ST_ ranged from 0.06 (BPPCT007) to 0.29 (BPPCT039), with a mean value of 0.13, showing low genetic differentiation due to the gene flow between accessions [29] (Table 1).

### 2.2. Genetic Relationships by UPGMA

The analysis for the detection of homonyms and synonyms allowed the identification of 141 unique genotypes and 2 synonyms: ‘IBG047’ and ‘IBG057’, both accessions from the Ibergen breeding program, which were grouped in the subgroup B6. 

**Figure 1:**
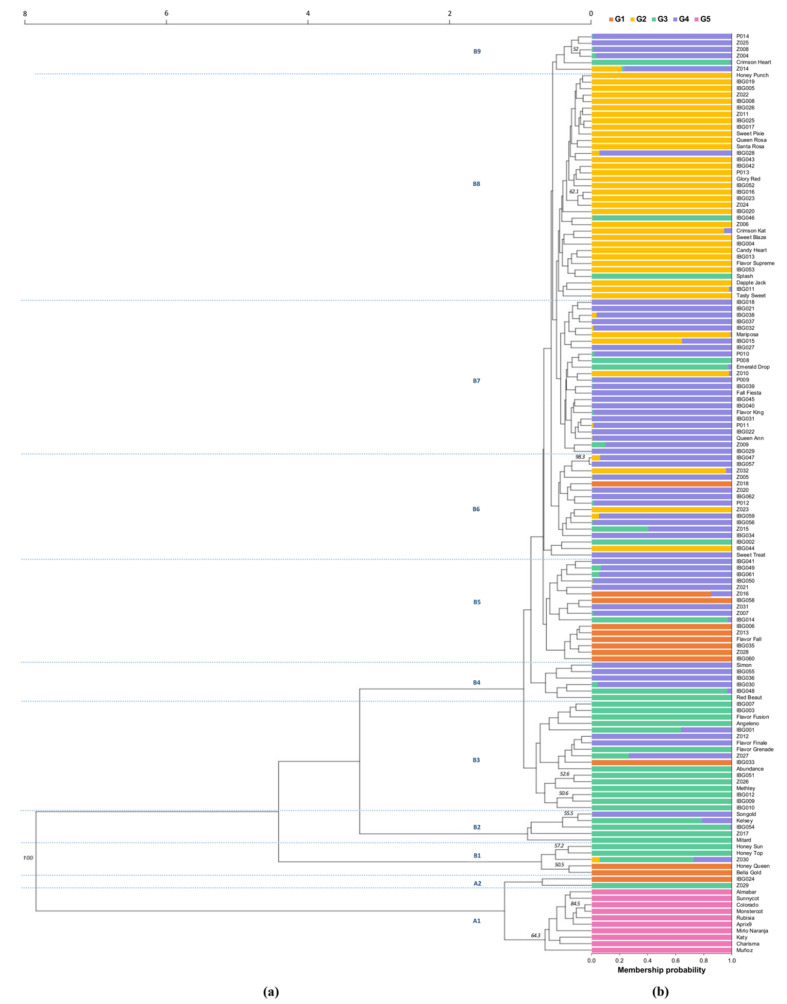
Genetic relationships and population structure from 115 interspecific plum × apricot hybrids and 27 reference genotypes (diploid plums and apricots). (**a**) The genetic relationships are represented by an unweighted pair group method based on arithmetic averages (UPGMA) dendrogram created from 1000 bootstrap replications. Bootstrap values >50% were placed on the branches. (**b**) The population structure is represented by the stacked bar chart. The *x*-axis provides the probability of each accession belonging to the assigned group identified by discriminant analysis of principal components (DAPC) (*K* = 5). These groups are depicted in orange (G1), yellow (G2), green (G3), purple (G4), and pink (G5).

The genetic relationships between the interspecific hybrids and the reference genotypes were assessed using the unweighted pair group method based on arithmetic averages (UPGMA) to generate a dendrogram based on the Nei and Li similarity index. The dendrogram showed the first node with a high bootstrap value (100), separating the accessions into two groups: group A, formed by 12 accessions (8.5%); and group B, formed by 130 accessions (91.5%) (Figure 1a). These two groups were divided into several internal secondary nodes. 

Group A was divided into two subgroups. The subgroup A1 was formed by the ten reference genotypes of apricot. In subgroup A2, two advanced selections from different breeding programs were grouped, ‘IBG024’ and ‘Z029’, which could be aprium hybrids since they showed a greater genetic relationship with the apricot reference genotypes than with those of plum [12]. 

Group B, the larger of the two groups, was made up of the rest of the interspecific hybrids and the reference genotypes of diploid plums (*P. cerasifera*, *P. salicina*, and *P. simonii*). Plumcots, pluots, and the reference genotypes were allocated into different groups, mixing and grouping mainly according to their genealogical origin. This grouping trend has been found between pluots, plumcots, plums, and apricots in a previous study, suggesting that the grouping is mainly due to the relationship between the accessions and their parents [20]. 

Subgroup B1 was separated a greater distance from the rest, grouping five Zaiger Genetics accessions. Two of them were reference genotypes: ‘Honey Top’, a nectarine (*P. persica*) with yellow flesh [30]; and ‘Honey Sun’, an apricot × peach hybrid [12]. The accessions ‘Honey Queen’, a yellow-fleshed nectarine, and ‘Bella Gold’, a peacotum [12], might have in their pedigree some genotype of *P. persica*. Subgroup B2 was a small but diverse group, grouping the reference genotypes ‘Kelsey’ (*P. salicina*), ‘Mitard’ (*P. cerasifera*), and ‘Songold’ (‘Golden King’ (unknown origin) × ‘Wickson’ (*P. salicina*)). ‘Songold’ could be a hybrid between *P. salicina* and *P. cerasifera* since it is grouped close to ‘Mitard’ (*P. cerasifera*). ‘Songold’ was developed by ARC-Infruitec, a public breeding program of South Africa that frequently uses ‘Methley’ (*P. salicina* × *P. cerasifera*) as a parent [31]. In subgroup B3, ‘Methley’ was grouped with ‘Angeleno’ (‘Queen Ann’ × Unknown), ‘Abundance’ (*P. salicina*), and the pluots of Zaiger Genetics: ‘Flavor Fusion’, ‘Flavor Finale’, and ‘Flavor Grenade’. These accessions were located near to ‘Red Beaut’ (‘Burmosa’ × ‘Eldorado’) [31], which was allocated in subgroup B4 with ‘Simon’ (*P. simonii*). Subgroup B5 contained some commercial cultivars from Zaiger Genetics. In subgroup B6, 16 accessions were allocated, including the reference genotype ‘Sweet Treat’ (pluerry). 

‘Red Beaut’ is one of the cultivars most used by Zaiger Genetics as a parent [32], which could explain why Zaiger accessions were allocated in the same group B to which ‘Red Beaut’ and other cultivars usually used as parents such as ‘Queen Ann’ (‘Gaviota’ × ‘Eldorado’) and ‘Mariposa’ (*P. salicina*) [33,34,35]. ‘Queen Ann’ and ‘Mariposa’ were grouped in subgroup B7 with three pluots (‘Emerald Drop’, ‘Fall Fiesta’, and ‘Flavor King’). The subgroup B8 was the most numerous of all (*n* = 35), including four reference genotypes (‘Dapple Jack’, ‘Queen Rosa’, ‘Santa Rosa’, and ‘Splash’) and eight commercial cultivars. The genealogy of ‘Glory Red’ includes ‘Queen Ann’ [32], which was used as a parent of ‘Queen Rosa’ in a cross with ‘Santa Rosa’ [31]. The three cultivars ‘Glory Red’, ‘Queen Rosa’, and ‘Santa Rosa’ could therefore be genetically related. The pluot cultivars ‘Splash’ and ‘Dapple Jack’ were also genetically related, as ‘Splash’ is the ancestor of ‘Dapple Jack’ [32]. Finally, six accessions were included in subgroup B9. Subgroups B6 and B9 were made up of only interspecific hybrids, which possibly shared parents among them.

The advanced selections were allocated in the subgroups A2 and B1–B9, showing the same diversity observed among the commercial cultivars. 

### 2.3. Analysis of Population Structure by DAPC

**Figure 2:**
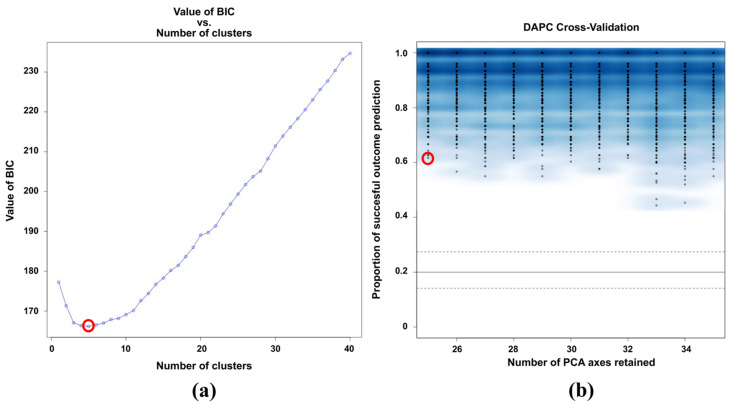
Clustering and DAPC cross-validation. (**a**) Inference of the optimal number of clusters in the 115 interspecific plum × apricot hybrids and 27 reference genotypes (diploid plums and apricots), and (**b**) DAPC cross-validation for the optimal number of principal components (PCs) retained for the analysis in the five predefined groups.

**Figure 3:**
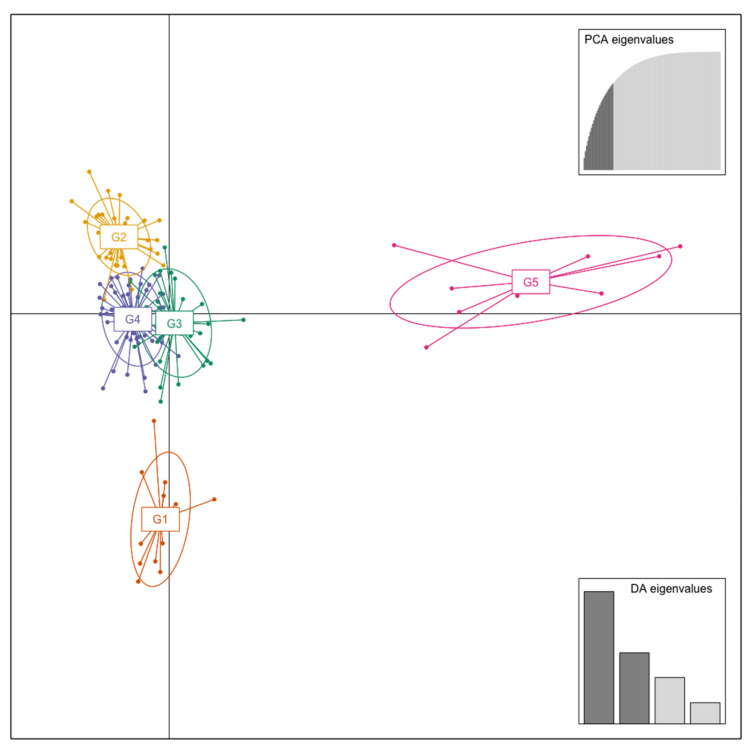
Scatterplot of discriminant analysis of principal components (DAPC) of population structure of 115 interspecific plum × apricot hybrids accessions and 27 reference genotypes (diploid plums and apricots) using eight SSR markers. The axes represent the first two linear discriminants (LD1 and LD2) according to the discriminant analysis functions (DA) eigenvalues. Four discriminant analysis functions (DA) eigenvalues and 25 principal component analysis (PCA) eigenvalues were retained during analyses to describe the relationship between the five groups. Each dot represents an accession, and each colored circle represents a group identified by DAPC analysis. G1 = group formed mainly by advanced selections (orange), G2 = group with more commercial cultivars (yellow), G3 = group with more reference genotypes (green), G4 = largest group (purple), and G5 = apricot group (pink).

To establish the pattern of the population structure, discriminant analysis of principal components (DAPC) was performed. Despite the high degree of introgression in this type of interspecific hybrids [2,7,12,34,36], the DAPC analysis showed the formation of five groups (*K* = 5) according to the lowest BIC value (166.17) (Figure 2A). The cross-validation of the DAPC showed that the proportion of success for the prediction of the groups (*K* = 5) would be obtained with 25 principal components (PCs) (Figure 2b), so 25 PCs (Figure 3—inset of PCA eigenvalues) and four eigenvalues of the discriminant analysis functions (DA) (Figure 3—inset of DA eigenvalues) were retained for the DAPC analysis.

In the scatterplot of the DAPC analysis (Figure 3), groups G2, G3, and G4 overlapped near the intersection of the first two linear discriminants (LD1 and LD2). Groups G1 and G5 differed from the rest of the groups through LD2 and LD1, respectively. The membership probabilities of each accession belonging to its assigned group are shown in Figure 1b and were based on the retained discriminant functions of the DAPC analysis. The stacked bars indicate the proportions of successful reassignment of accessions to their original groups. This grouping corresponded to the UPGMA dendrogram (Figure 1a), indicating that the accessions were grouped according to their genealogical origin. 

The accessions were allocated into five groups (*K* = 5) (Table S2 of the Supplementary Materials). Group G1 contained 13 accessions (9% of the total population), including three Zaiger Genetics commercial cultivars (‘Bella Gold’, ‘Flavor Fall’, and ‘Honey Queen’). In group G2 (*n* = 38, 27%), the reference genotypes ‘Dapple Jack’ (pluot) and ‘Mariposa’ (*P. salicina*) were grouped with ‘Queen Rosa’ and’ Santa Rosa’, which are considered simple hybrids of *P. salicina* [2]. Group G3 (*n* = 32, 23%) included reference genotypes of different species, such as ‘Abundance’ and ‘Kelsey’ (*P. salicina*), ‘Honey Sun’ and ‘Honey Top’ (*P. persica*), and ‘Mitard’ (*P. ceracifera*), and some hybrids of *P. salicina*, such as ‘Angeleno’, ‘Methley’ (*P. salicina* × *P. cerasifera*), and ‘Red Beaut’. Group G4 included the largest number of accessions (*n* = 49, 35%), including four reference genotypes: ‘Queen Ann’, ‘Simon’ (*P. simonii*), ‘Songold’, and ‘Sweet Treat’ (pluerry). Finally, group G5 was formed exclusively by the 10 reference genotypes of *P. armeniaca* (apricot). 

The composition of groups G1, G2, G3, and G4 was homogeneous, probably due to their accessions being obtained from the selection of crosses and backcrosses in which common parents such as ‘Mariposa’, ‘Queen Ann’, ‘Queen Rosa’, ‘Friar’, and ‘Red Beaut’ were used [20,31,37].

### 2.4. Genetic Diversity among Groups by AMOVA

The analysis of molecular variance (AMOVA) on genetic differentiation among the five groups based on DAPC and within accessions revealed that 80% of the total variation in the genetic structure (*K* = 5) was attributed to the variability within accessions with significant differences (*p* < 0.01) (Table 2), a percentage similar to that observed in a population of Japanese plums (81.8%) in a previous study [15]. The variance among the five groups inferred with the DAPC analysis represented 11% of the total, and the variance among accessions within the five inferred groups represented the remaining 9%. Previous studies on apricot [25] and almond [38] reported that the variance between groups contributed 8 and 29% of the total variance, respectively, being much lower than the variance due to differences between the accessions, which corresponds with the results obtained in this work.

The parameters of genetic diversity were calculated for each of the five groups (*K* = 5) (Table 3). The number of alleles per locus (*N*_A PER LOCUS_) ranged from 6 (G5) to 12 (G3). The total number of alleles for each group ranged from 49 (G5) to 95 (G3). The same trend was observed in allelic richness (*A*_R_), with values between 6.25 (G5) and 8.14 (G3). Alleles observed in only one group were considered private alleles (*P*_A_), with the smallest value (4) in group G2 and the largest value (24) in group G5. These results showed moderate and relatively homogeneous levels of genetic diversity, except in group G3, which showed a greater number of alleles (*N*_A PER LOCUS_ and *N*_A TOTAL_), greater allelic richness (*A*_R_), and a value of the coefficient of inbreeding notably higher than the rest (*F_I_*_S_ = 0.21), revealing a heterozygosity deficit, as observed in a diverse group of apricots from different geographical origins [25]. The values of observed heterozygosity (*H*o) ranged from 0.61 (G2) to 0.75 (G1 and G5), and the expected heterozygosity (*H*e), from 0.67 (G2) to 0.86 (G3). *H*o was slightly lower than *He* in groups G1, G2, G3, and G4, which could be attributed to the exhaustive breeding activity developed in these hybrids. The heterozygosity (*Ho* and *He*) of G5, which includes the apricot cultivars, was similar to that observed in a previous work including traditional and new apricot cultivars [26].

**Figure 4:**
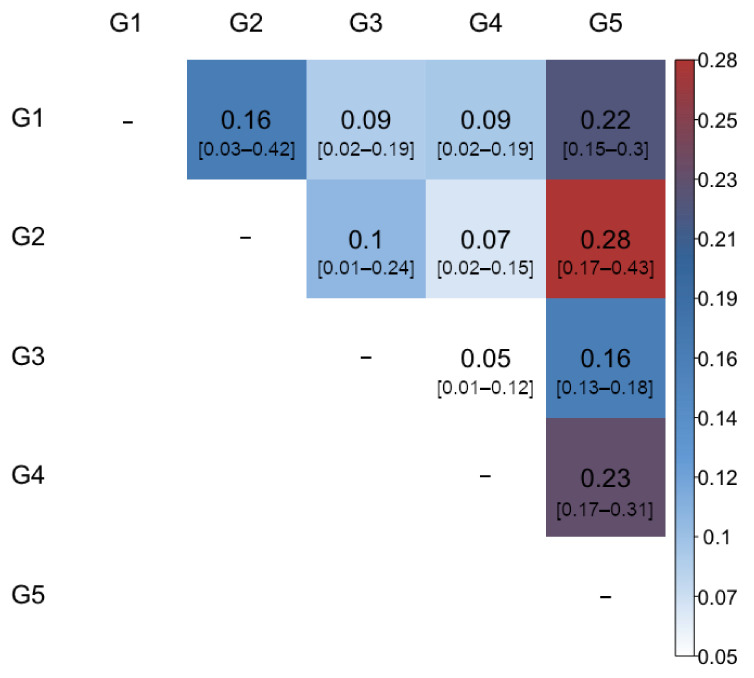
Correlation plot of pairwise genetic differentiation values (*FST*) among the five groups inferred by DAPC (*K* = 5). Lower and upper limits of the 99% confidence interval based on 1000 bootstrap replicates are shown in brackets.

To validate the genetic differentiation between groups, the correlations of pairwise genetic differentiation values (*F_ST_*) were determined (Figure 4). The mean value observed was 0.16 and ranged from 0.05 (between G3 and G4) to 0.28 (between G2 and G5), indicating a moderate differentiation between groups. The correlations between group G5, which was formed entirely by apricot cultivars, and the rest of the groups showed the highest values, revealing a restricted flow of genes from apricot cultivars towards the 132 accessions that were grouped in G1 to G4 (diploid plums, plumcots, pluots, and other hybrids). A moderate but significant genetic differentiation was observed in G1, G2, G3, and G4, except the correlation between groups G3 and G4, which showed slight genetic differentiation. All the correlations presented lower and upper limits different from zero within a 99% confidence interval (Figure 4). 

Since their introduction at the beginning of the 20th century, the interspecific plum × apricot hybrids have generated controversy due to the lack of clarity in classifying them as plum, plumcot, pluot, or aprium for their commercialization, due to the similarity in the appearance of their fruits, as well as the difficulty in determining the quality standards that must be applied [7]. The DAPC analysis used to assess the population structure revealed the five groups in which the whole population was structured. This structure is related with the genealogical background of the accessions, which can be useful for inferring the real interspecific status of the complex interspecific plum × apricot hybrids analyzed. The ability to distinguish these hybrids is not only important to sellers and consumers but also to breeders [8] and producers [20], due to the different agronomic management. In certain markets, such as the USA, the classification as plums or pluots can greatly affect the price of the fruit [9]. 

The four inferred groups including the interspecific hybrids showed a weaker genetic relationship to the apricot group than would be expected if they were simple plum × apricot hybrids, as suggested by the terms ‘plumcot’, ‘pluot’, and ‘aprium’ [12]. A closer genetic relationship of pluots to Japanese plums than to apricots has also been found in a previous study [20]. Previous analysis of the genetic structure and diversity between interspecific hybrids is limited to a study that included 29 Japanese plum cultivars, 4 interspecific hybrids, 2 cultivars of *P. domestica*, a cultivar of *P. cerasifera*, and another of *P. armeniaca*, which showed a low genetic differentiation between the determined population structure [35]. The unexpected distance found between interspecific hybrids and apricot cultivars may be due to several backcrosses, or new crosses with plum cultivars, of the first descendants of simple plum × apricot hybrids, to search for fruits more similar to glabrous-skinned plums than to apricots [13]. 

Although many species are involved in the genealogy of the interspecific hybrids, our results suggest that the observed diversity is lower than expected, probably due to the use of the same parents, such as ‘Friar’, ‘Mariposa’, ‘Queen Ann’, ‘Queen Rosa’, and ‘Red Beaut’, in different breeding programs [32]. In addition, the Japanese plum cultivars used as parents are complex hybrids that come directly or indirectly from the first hybridizations carried out by Luther Burbank, who used as parents a small number of Japanese plum cultivars (‘Abundance’, ‘Burbank’, ‘Kelsey’, and ‘Satsuma’) [4], other diploid plums (‘Maritima’ (*P. maritima*), ‘Simon’ (*P. simonii*), and ‘Robinson’ (*P. munsoniana*)), and the first simple hybrids (‘Gaviota’, ‘Santa Rosa’, and ‘Wickson’) [5]. In previous works, low percentages of fruit set have been obtained in plum × apricot crosses, and very low or even null in apricot × plum crosses, which shows the difficulty of obtaining these hybrids [10,11]. This situation may have caused some of the hybrids to have been erroneously considered as plumcots, pluots, or apriums, being Japanese plum-type hybrids. However, it is difficult to determine the genealogy of the interspecific hybrids, since in most cases the parents are unknown.

## 3. Materials and Methods

### 3.1. Plant Material

A group of 142 accessions was analyzed, including 27 reference genotypes (Table 4), 18 commercial cultivars (Table 5), and 97 advanced selections of interspecific hybrids from three breeding programs: Ibergen (62 accessions, IBG001-IBG062), Provedo (7 accessions, P008-P014), and Zaiger Genetics (46 accessions, Z004-Z032). The plant material of the interspecific hybrids was obtained from several experimental orchards: Ibergen, located in Caspe (Zaragoza, Spain) and San Rafael del Río (Castellón, Spain) (72 accessions); Viveros Mariano Soria, in La Almunia de Doña Godina (Zaragoza, Spain) (36 accessions); and Provedo, in Don Benito (Badajoz, Spain) (7 accessions) (Table 4 and Table 5). The plant material of the 27 reference genotypes was obtained from the germplasm collections of Centro de Investigaciones Científicas y Tecnológicas de Extremadura (CICYTEX-La Orden) in Guadajira (Badajoz, Spain) (4 genotypes), Centro de Investigación y Tecnología Agroalimentaria de Aragón (CITA) in Zaragoza (Zaragoza, Spain) (14 genotypes) and Viveros Mariano Soria in La Almunia de Doña Godina (Zaragoza, Spain) (9 genotypes) (Table 5). 

### 3.2. DNA Extraction

For each accession, DNA was extracted from young leaves collected in spring and preserved in silica gel [39]. Once dried, the leaf tissue was ground on a TissueLyser (Qiagen, Hilden, Germany). Genomic DNA extraction was carried out by using a Speedtools Plant DNA Extraction Kit (Biotools, Madrid, Spain) [40,41,42,43] following the protocol described by Hormaza [24]. The quantity and quality of each DNA sample were determined using a spectrophotometer, NanoDrop 1000 (ThermoScientific, Waltham, MA, USA). DNA of each accession was diluted to 10 ng/μL and stored at −20 °C until PCR amplification [40].

### 3.3. SSR Genotyping

For SSR analysis, eight SSR markers were selected from the previously reported studies in Japanese plum [44] and peach [45,46,47,48] (Table 6). The SSR markers used in this study have shown high transferability among different *Prunus* species in previous reports [15,20,24,25].

The amplification was performed using five sets of multiplex PCR reactions (M01 to M05). Each multiplex reaction was designed according to the protocol described by Guerrero et al. [15]. All reactions were performed with 10 ng of genomic DNA, different concentrations for each SSR marker (Table 6), and 1X Qiagen Multiplex PCR Master Mix (Qiagen, Hilden, Germany) and a SimplyAmp Thermal Cycler (Applied Biosystems, Foster City, CA, USA). A final volume of 12.5 µL was used in Multiplex PCR reactions M01–M03, and a final volume of 11.5 µL in M04 and M05. The amplification was performed with the following cycles: in M01 to M03, the temperature profile used had an initial denaturation step at 95 °C for 15 min, followed by 35 cycles at 95 °C for 45 s, 57 °C for 45 s, 72 °C for 2 min, and a final step at 72 °C for 30 min [46]. The same conditions were used for M04 and M05 but modifying the annealing temperature at 46 °C and 62 °C, respectively [44]. The amplicons were separated by capillary electrophoresis using a genetic analyzer ABI3730 (Applied Biosystems, Foster City, CA, USA). A size standard GeneScan 500LIZ (Applied Biosystems, Foster City, CA, USA) was used to estimate the molecular size (pb) of the amplicons that were scored on the R [49] package for fragment analysis ‘Fragman’ [50], and confirmed with the software PeakScanner v. 1.0 (Applied Biosystems, Foster City, CA, USA) (Supplementary Materials, Figure S1). Before the data analysis, the genetic profiles were organized on a table in CSV format (Supplementary Materials, Table S1).

### 3.4. Data Analysis

The data of alleles generated by the SSR markers were converted into an object of the class ‘genind’ using the ‘df2genind’ function of the ‘adegenet’ package v. 2.1.2. [51] and analyzed with the R software v 3.6.0 [49]. 

#### 3.4.1. Genetic Diversity Analysis

The genetic diversity parameters (number of alleles per locus *(N*_A_), polymorphism information content (*PIC*), private alleles (*P_A_*), allelic richness (*A*_R_), observed heterozygosity (*H*o), expected heterozygosity (*H*e), inbreeding coefficient (*F*_IS_), and Wright’s fixation index (*F*_ST_)) were determined on the whole population using the packages ‘adegenet’ v. 2.1.2 [51], ‘hierfstat’ v. 0.5-7 [52], ‘pegas’ v. 0.13 [53] and ‘PopGenReport’ v. 3.0.4 [54]. 

#### 3.4.2. Detection of Homonymies and Synonymies

The sizes of the alleles were compared by the ‘duplicated’ function to detect identical genetic profiles, which were considered as synonymies, and the accession names to detect identical names with different genetic profiles, which were considered as homonymies [15,26].

#### 3.4.3. Establishment of Genetic Relationships

An unweighted pair group method with arithmetic averages (UPGMA) cluster analysis was used to determine the genetic relationships among accessions according to Nei and Li [55]; a UPGMA dendrogram with a ‘bootstrap’ supported by 1000 replicates was generated with the ‘poppr’ package v. 2.8.5 [56]. 

#### 3.4.4. Determination of Population Structure

The population structure was analyzed by a discriminant analysis of principal components (DAPC) using the ‘adegenet’ package v. 2.1.2. [51,57]. The optimal number of groups (*K*) in the whole population was inferred using the ‘find.clusters’ function and according to the lowest Bayesian information criterion (BIC) value. The correct numbers of principal components (PCs) to be retained were determined using the cross-validation function ‘xvalDapc’. The membership probabilities were obtained from the DAPC objects in the slot posterior (‘class(dapc1$posterior)’). The slot ‘assign.per.pop’ was used to indicate the proportions of successful reassignment (based on the discriminant functions) of accessions to their original groups [57]. 

The genetic diversity analysis at the group level was performed using the same parameters determined in the whole population (3.4.1.). The package ‘hierfstat’ v. 0.5-7 [52] was used to calculate the correlation matrix of the pairwise F_ST_ values using the ‘pp.fst’ function. 

Finally, the variance components among the inferred groups and the accessions were calculated with an analysis of molecular variance (AMOVA) using the ‘poppr’ package v. 2.8.5 [56].

## 4. Conclusions

The molecular characterization allowed structuring the interspecific plum × apricot hybrids into five groups that clearly corresponded with the genealogical background. The growing interest in obtaining new interspecific hybrids of *Prunus* due to their commercial potential [16] led to the fact that most of these accessions have been obtained after several backcrosses with plum [2,12], which would explain their greater genetic proximity to plum than to apricot. This genetic relationship corresponds to the fruit morphology and agronomic behavior observed in most interspecific hybrids, which also resemble plums more than apricots. The low genetic diversity found herein could be related to the repeated use of a small group of parents and the inbreeding produced by backcrosses during the breeding process, as it has been observed in apricot [26] and Japanese plum-type cultivars [15]. These results can be useful for the management of germplasm repositories in order to avoid the loss of genetic diversity and the selection of parents for breeding purposes. Further studies using other approaches such as high-throughput genotyping-by-sequencing (GBS) can be useful for mapping and for the detection of trait-associated QTLs to continue the exploitation of this material.

## Figures and Tables

**Table 1:** Genetic diversity parameters of 115 accessions of interspecific plum × apricot hybrids and 27 reference genotypes using eight SSR markers. Number of alleles (*N_A_*), allele size, polymorphism information content (PIC), observed heterozygosity (*Ho*), expected heterozygosity (*He*), inbreeding coefficient (*F_IS_*), and Wright’s fixation index (*F_ST_*).

Locus	*N*_A_	Allele Size (bp)	PIC	*H*o	*H*e	*F*_IS_	*F*_ST_
pchgms2	20	128–170	0.84	0.85	0.83	−0.03	0.07
CPPCT033	12	122–159	0.61	0.66	0.58	−0.13	0.21
BPPCT007	21	113–167	0.80	0.81	0.82	0.01	0.06
BPPCT039	20	121–173	0.78	0.48	0.60	0.20	0.29
BPPCT025	18	148–196	0.82	0.73	0.78	0.07	0.09
CPSCT026	17	156–210	0.87	0.49	0.78	0.37	0.14
CPSCT005	20	167–229	0.90	0.73	0.87	0.16	0.07
UDP96005	21	95–73	0.85	0.83	0.83	0.00	0.08
*Mean*	*19*	*-*	*0.81*	*0.70*	*0.76*	*0.08*	*0.13*

**Table 2:** Analysis of molecular variance (AMOVA) for 115 interspecific plum × apricot hybrids and 27 reference genotypes (diploid plums and apricots) structured in five groups (*K* = 5).

Source of Variation	df	Sum of Square	Mean Sum of Square	% of the Variance	Phi
Among groups (*K* = 5)	4	187	47	11	0.20
Among accessions within groups (*K* = 5)	137	913	7	9	0.10
Within accessions	142	772	5	80 *	0.11
Total	283	1872	7	100	-

* Significant values at *p* < 0.01 significance level.

**Table 3:** Parameters of genetic diversity of the genetic structure (*K* = 5) of 115 accessions of interspecific hybrids and 27 reference genotypes using eight SSR markers. Number of alleles (*N_A_*), allele richness (*A_R_*), private alleles (*P_A_*), observed heterozygosity (*Ho*), expected heterozygosity (*He*), and inbreeding coefficient (*F_IS_*).

Group	*n*	*N*_A PER LOCUS_	*N*_A TOTAL_	*A*_R_	*P*_A_	*H*o	*H*e	*F_I_*_S_
G1	13	9	69	7.75	11	0.75	0.78	0.04
G2	38	9	75	6.82	4	0.61	0.67	0.06
G3	32	12	95	8.14	8	0.68	0.86	0.21
G4	49	10	82	6.94	5	0.70	0.76	0.05
G5	10	6	49	6.25	24	0.75	0.75	−0.01

**Table 4:** Reference genotypes analyzed in this study.

Reference Genotypes	
Almabar ^4^ (*P. armeniaca*)	Honey Top ^15^ (*P. persica*)
Aprix9 ^8^ (*P. armeniaca*)	Kelsey ^17^ (*P. salicina*)
Charisma ^1^ (*P. armeniaca*)	Mariposa ^2^ (*P. salicina*)
Colorado ^9^ (*P. armeniaca*)	Methley ^7^ (*P. salicina* × *P. cerasifera*)
Katy ^15^ (*P. armeniaca*)	Mitard ^16^ (*P. cerasifera*)
Mirlo Naranja ^3^ (*P. armeniaca*)	Queen Ann ^14^ (Gaviota × Eldorado)
Monstercot ^13^ (*P. armeniaca*)	Queen Rosa ^14^ (Queen Ann × Santa Rosa)
Muñoz ^16^ (*P. armeniaca*)	Red Beaut ^10^ (Burmosa × Eldorado)
Rubisia ^6^ (*P. armeniaca*)	Santa Rosa ^7^ (*P. salicina*)
Sunnycot ^11^ (*P. armeniaca*)	Simon ^12^ (*P. simonii*)
Abundance ^17^ (*P. salicina*)	Songold ^1^ (Golden King × Wickson)
Angeleno ^5^ (‘Queen Ann’ x unknown)	Splash ^15^ (Pluot)
Dapple Jack ^15^ (Pluot)	Sweet Treat ^15^ (Pluerry)
Honey Sun ^15^ (*P. persica × P. armeniaca*)	

^1^ ARC-Infruitech, South Africa; ^2^ Armstrong Nursery, USA; ^3^ CEBAS-CSIC, Spain; ^4^ Frutaria, Spain; ^5^ Garabedian, USA; ^6^ IPS, France; ^7^ Luther Burbank, USA; ^8^ Proseplan, Spain; ^9^ PSB Producción Vegetal, Spain; ^10^ Reedley Nursery, USA; ^11^ SDR FRUIT LLC, USA; ^12^ Simon Brothers, USA; ^13^ SMS Unlimited, USA; ^14^ USDA, USA; ^15^ Zaiger Genetics, USA; ^16^ unknown; ^17^ imported from Japan by L. Burbank.

**Table 5:** Commercial cultivars of interspecific hybrids of Zaiger Genetics analyzed in this study and their available pedigree information.

Commercial Cultivars	Available Pedigree Information
Bella Gold	Geo Pride, Flavor Queen, Mariposa, Red Beaut
Candy Heart	Autumn Giant, Black Kat, Red Beaut
Crimson Heart	Friar, Flavorosa, Laroda, Plum Parfait, Queen Ann
Crimson Kat	Flaming Gold, Flavor Treat, Mariposa, Red Beaut
Emerald Drop	Friar, Flavor Queen, Red Beaut
Fall Fiesta	Dapple Fire
Flavor Fall	Unknown
Flavor Finale	Casselman, King David, Queen Ann, Red Beaut
Flavor Fusion	Bella Sun, Con-N-Candy, Poppy, Red Beaut
Flavor Grenade	Flavor Queen, Mariposa, Red Beaut
Flavor King	Flavor Queen, Mariposa, Red Beaut
Flavor Supreme	Red Beaut
Glory Red	Burmosa, Flavor Fall, Mariposa, Red Beaut
Honey Punch	Autumn Giant, Friar, Modesto, Red Beaut, Splash
Honey Queen	Unknown
Sweet Blaze	Unknown
Sweet Pixie	*P. avium* (Bing, Nadia, Royal Lee, Stella)
Tasty Sweet	Unknown

**Table 6:** Multiplex (Mp) design, SSR loci, linkage group (LG), fluorescent dyes, primer concentration (PC), PCR details and characteristics of the eight SSR markers analyzed in this study.

Mp	Locus	LG	Dye	PC (µM)	Primer Sequence	SSR Motif	Size Range (bp)	Species
M01	pchgms2 [45]	G4	6-FAM	0.2	F: GTCAATGAGTTCAGTGTTACACTC	(CT)_24_	130–200	Peach
					R: AATCATAACATCATTCAGCCACTGC			
	CPPCT033 [48]	G7	NED	0.2	F: TCAGCAAACTAGAAACAAACC	(CT)_16_	151	Peach
					R: TTGCAATCTGGTTGATGTT			
M02	BPPCT-007 [46]	G3	6-FAM	0.2	F: TCATTGCTCGTCATCAGC	(AG)_22_(CG)_2_(AG)_4_	143–151	Peach
					R: CAGATTTCTGAAGTTAGCGGTA			
	UDP96-005 [47]	G1	VIC	0.3	F: GTAACGCTCGCTACCACAAA	(AC)_16_TG(CT)_2_CA(CT)_11_	100–250	Peach
					R: CCTGCATATCACCACCCAG			
M03	BPPCT-039 [46]	G3	PET	0.3	F: ATTACGTACCCTAAAGCTTCTGC	(GA)_20_	148–158	Peach
					R: GATGTCATGAAGATTGGAGAGG			
	BPPCT-025 [46]	G6	VIC	0.3	F: TCCTGCGTAGAAGAAGGTAGC	(GA)_29_	178–202	Peach
					R: CGACATAAAGTCCAAATGGC			
M04	CPSCT026 [44]	G7	6-FAM	0.3	F: TCTCACACGCTTTCGTCAAC	(CT)_16_	177–213	Japanese plum
					R: AAAAAGCCAAAAGGGGTTGT			
M05	CPSCT005 [44]	G4	NED	0.3	F: CTGCAAGCACTGCGGATCTC	(CT)_15_	171–191	Japanese plum
					R: CCCATATTCCCAACCCATTA			

## Data Availability

Not applicable.

## Supplementary Materials

The following are available online at https://www.mdpi.com/article/10.3390/plants11091241/s1. Figure S1: Representative electropherogram profiles of the fluorescence-labeled simple sequence repeat (SSR) products amplified with the pair of primer CPSCT026. (a) Japanese plum (*Prunus salicina*) cultivar ’Kelsey’. (b) Interspecific hybrid cultivar (pluot) ‘Flavor King’. (c) Apricot (*Prunus armeniaca*) cultivar ‘Charisma’. Table S1: Genotypes of 115 accessions of interspecific plum × apricot hybrids and 27 reference genotypes using eight SSR markers. Table S2: Accessions and group assignment performed by DAPC (*K* = 5).
