# Supplementary material for: Simple Sequence Repeat (SSR)-Based Genetic Diversity in Interspecific Plumcot-Type (Prunus salicina × Prunus armeniaca) Hybrids
Source: Plants (Basel). 2022 May 4;11(9):1241. doi: 10.3390/plants11091241 (PMC9105337; doi:10.3390/plants11091241)
Supplement: Supplementary file 1 [file plants-11-01241-s001.zip › Supplementary Materials_R2/Figure S1 .pdf]

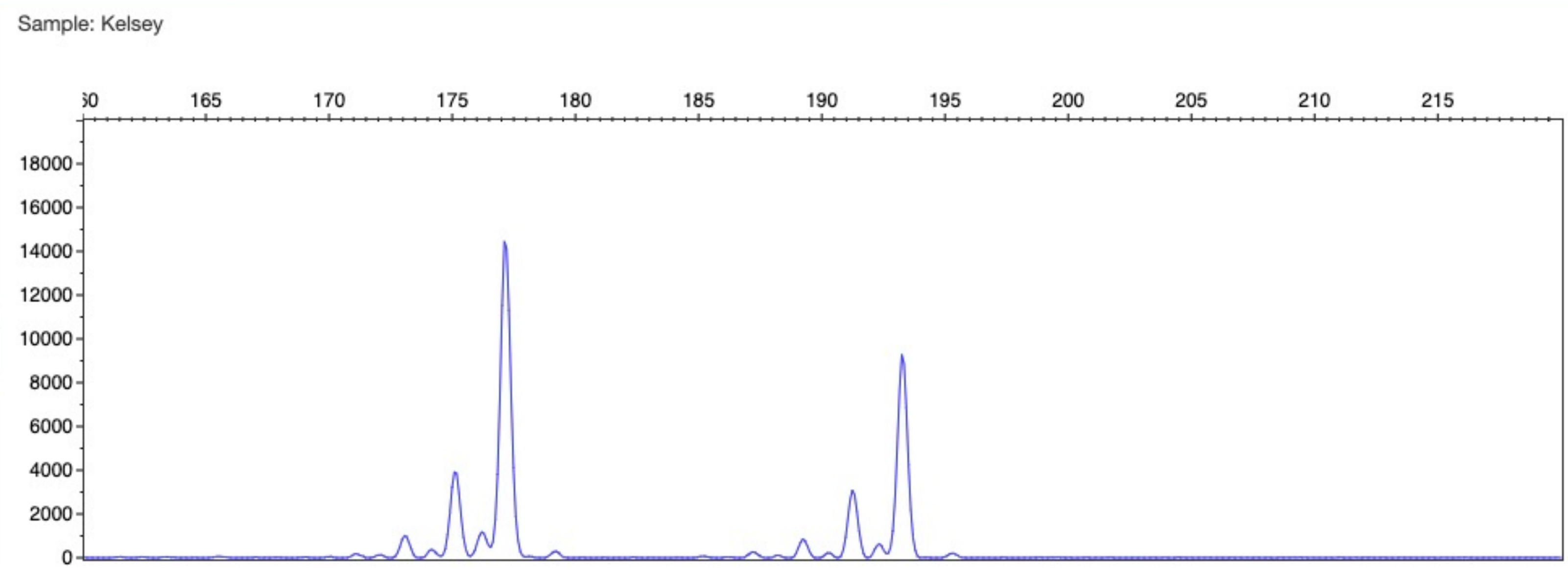

(a)

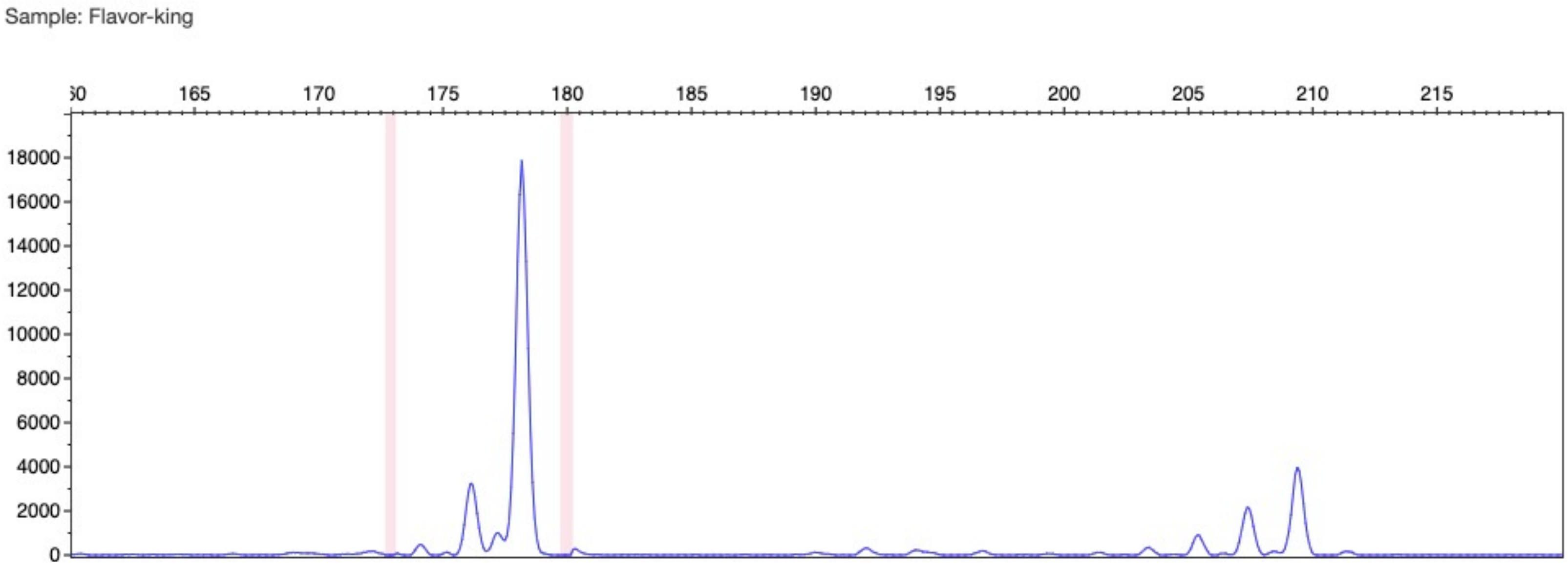

(b)

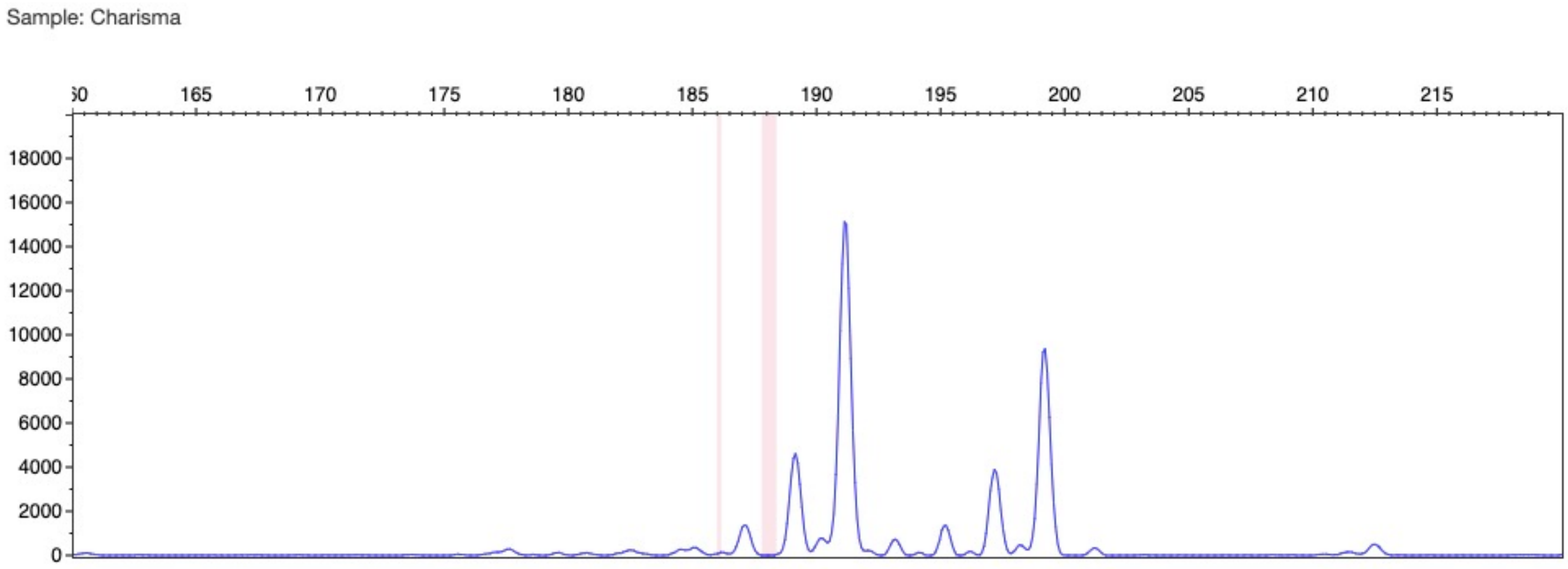

(c)

Figure S1. Representative electropherogram profiles of the fluorescence-labeled simple sequence repeat (SSR) products amplified with the pair of primer CPSCT026. (a) Japanese plum (*Prunus salicina*) cultivar 'Kelsey'. (b) Interspecific hybrid cultivar (pluot) 'Flavor King'. (c) Apricot (*Prunus armeniaca*) cultivar 'Charisma'.
